# The Proprietary Medicines Bill

**Published:** 1920-08-07

**Authors:** 


### THE PROPRIETARY MEDICINES BILL.

The report of the Select Committee of the House of Commons, presided over by Sir Henry Norman, on "patent medicines" was dated August 4, 1914 —a date which is more than sufficiently familiar in another connection. Not enough public attention was given at the time to the Norman Report, which was itself a most attractively written document, full of "wise saws and modern instances," and especially praiseworthy by reason of the level-headed discrimination of its authors between the legitimate and fraudulent groups of proprietary medicines.

It has remained for Dr. Addison, Viscount Astor, and their advisers to take the first step towards translating the greater number of the Select Committee's recommendations into law. The Proprietary Medicines Bill, just introduced in the House of Lords by Viscount Astor, gives effect to the more important of those recommendations, but so far only as they fall within the province of the Ministry of Health. Before considering these it will be well to see which of the Norman recommendations have been ruled out as beyond Dr. Addison's province. There is, first, the important recommendation that the administration of the law governing the advertisement and sale of secret and patent medicines should be co-ordinated and combined under one State Department. Reference to the report of the Select Committee shows that, in their opinion, certainly the powers and duties of the Privy Council in relation to the Pharmacy Acts, and perhaps also those of other Departments in other respects, should be transferred to the Ministry of Health. Viscount Astor and his chief have not ventured in this Bill to propose such a transfer, but he would be a bold man who would prophesy that such a change will not before long be effected. Nor does the new Bill deal at all with the anomalies of the Medicine Stamp Duty Acts, while the proposal to establish a special court as a judicial authority with powers to prohibit the sale or advertisement of particular preparations, has similarly been deemed to be foreign to the province of the Ministry of Health, and the question of fixing a limit to the validity of trade-marks used as trade names for drugs is also not dealt with by the Bill.

With these exceptions the Bill follows very closely the recommendations of Sir Henry Norman's Committee. "Disclosure by label" is not to be demanded, except when required by the Minister in the case of poisonous and dangerous or alcoholic ingredients. Provision is made for the establishment of a Register of proprietary medicines and surgical appliances and of the owners thereof, and the sale of an unregistered article or of an article placed on the market by an unregistered manufacturer is to be an offence. Formulas of medicines and patterns of appliances are to be revealed to and deposited with the Registrar, who will be an official of the Ministry of Health. The Registrar is forbidden, under heavy penalties, to divulge the constitution of any medicine. The sale is prohibited of remedies purporting to cure twelve scheduled diseases (cancer, consumption, lupus, deafness, fits, epilepsy, amenorrhœa and other diseases peculiar to women, diabetes, paralysis, locomotor ataxy, Bright's disease, and rupture). This list of diseases may be added to by the Minister of Health. Medical treatment by correspondence will not be allowed on the part of the proprietor of a secret remedy or proprietary surgical appliance. Advertisements of proprietary medicines and appliances will be strictly controlled. Falsity in certain respects will apparently be permitted, but bogus testimonials, alleged to have been given by medical practitioners, and lying statements about the medical origin of a remedy will be forbidden. The disclosure by label of the alcoholic content may be insisted on, as well as that of "any poisonous or dangerous drug." The therapeutic value claimed for the remedy may be required to correspond with that specified in the Register. Medicines or appliances which the Minister thinks injurious to health may be removed by him from the Register; in other words, he may prohibit sale; but an aggrieved owner may appeal to the High Court against such an order of removal from the Register. Existing stocks may be disposed of by all concerned, and another saving clause exempts all sales which may be made on the prescription of a qualified medical or dental practitioner. No authority is specifically named as the authority to enforce the Act, but presumably enforcement will be undertaken as a direct duty by the Registrar of Proprietary Medicines or other officials of the Ministry of Health.

